# Heterogeneous nutrient supply modulates root exudation and accumulation of medicinally valuable compounds in *Artemisia annua* and *Hypericum perforatum*

**DOI:** 10.3389/fpls.2023.1174151

**Published:** 2023-06-02

**Authors:** Martina Paponov, Juanita Flate, Jörg Ziegler, Cathrine Lillo, Ivan A. Paponov

**Affiliations:** ^1^ Division of Food Production and Society, Norwegian Institute of Bioeconomy Resesarch (NIBIO), As, Norway; ^2^ Department of Chemistry, Bioscience and Environmental Engineering, University of Stavanger, Stavanger, Norway; ^3^ Department of Molecular Signal Processing, Leibniz Institute of Plant Biochemistry, Halle, Germany; ^4^ Department of Food Science, Aarhus University, Aarhus, Denmark

**Keywords:** *Artemisia annua*, *Hypericum perforatum*, split-root system, heterogeneous nutrient supply, nutrient deficiency, nitrogen, phosphorus, iron

## Abstract

Plants have evolved complex mechanisms to adapt to nutrient-deficient environments, including stimulating lateral root proliferation into local soil patches with high nutrient content in response to heterogeneous nutrient distribution. Despite the widespread occurrence of this phenomenon in soil, the effect of heterogeneous nutrient distribution on the accumulation of secondary compounds in plant biomass and their exudation by roots remains largely unknown. This study aims to fill this critical knowledge gap by investigating how deficiency and unequal distributions of nitrogen (N), phosphorus (P), and iron (Fe) affect plant growth and accumulation of the antimalarial drug artemisinin (AN) in leaves and roots of *Artemisia annua*, as well as AN exudation by roots. Heterogeneous N and P supplies strongly increased root exudation of AN in half of a split-root system exposed to nutrient deficiency. By contrast, exposure to a homogeneous nitrate and phosphate deficiency did not modulate root exudation of AN. This indicates that a combination of local and systemic signals, reflecting low and high nutritional statuses, respectively, were required to enhance AN exudation. This exudation response was independent of the regulation of root hair formation, which was predominantly modulated by the local signal. In contrast to the heterogeneous supply of N and P, heterogeneous Fe supply did not modulate AN root exudation but increased AN accumulation in locally Fe-deficient roots. No modulation of nutrient supply significantly changed the accumulation of AN in *A. annua* leaves. The impact of a heterogeneous nitrate supply on growth and phytochemical composition was also investigated in *Hypericum perforatum* plants. Unlike in *A. annue*, the uneven N supply did not significantly influence the exudation of secondary compounds in the roots of *H. perforatum*. However, it did enhance the accumulation of several biologically active compounds, such as hypericin, catechin, and rutin isomers, in the leaves of *H. perforatum*. We propose that the capacity of plants to induce the accumulation and/or differential exudation of secondary compounds under heterogeneous nutrient supply is both species- and compound-specific. The ability to differentially exude AN may contribute to *A. annua*’s adaptation to nutrient disturbances and modulate allelopathic and symbiotic interactions in the rhizosphere.

## 1 Introduction

As sessile organisms, plants have evolved numerous mechanisms that allow them to adapt to otherwise unavoidable environmental changes. These mechanisms include the accumulation of secondary metabolites in plant tissues and their secretion into the rhizosphere. These secondary compounds play many roles, including defense against herbivory and microbial infection, reduction of damage from reactive oxygen species, regulation of development, and mediation of organismal interactions (i.e., allelopathic suppression of competitors, symbiosis with nitrogen-fixing bacteria and mycorrhiza, etc.). Taxon-specific biosynthesis of various classes of secondary metabolites therefore defines a plant’s fitness to specific ecological niches. Plants that synthesize many of these active compounds also represent a special interest as harvestable crops because, while these compounds can improve plant defenses and therefore their growth under stress, many are also widely used as medicines by humans (Yeshi et al., 2022).

Plants are often chosen for research based on their potential to produce valuable phytochemicals that can be used in developing new drugs and treatments of various health conditions. Among these plants, *Artemisia annua* and *Hypericum perforatum* are particularly noteworthy for their production of highly valuable phytochemicals. *A. annua* produces artemisinin, a sesquiterpene lactone and a powerful antimalarial drug that is considered the most effective treatment for malaria (Chadwick et al., 2013). *H. perforatum* produces antidepressant naphthodianthrones (NDAs) (hypericin and pseudohypericin), flavonoids, and other phenolic antioxidants (Mir et al., 2019). Our previous investigation have shown that light exposure of roots, which leads to increased production of reactive oxygen species (ROS), promotes the accumulation of phytochemicals in the aboveground parts of both medicinal plants, *A. annua* and *H. perforatum* (Paponov et al., 2023). However, the impact of other external stresses on accumulation of phytochemicals in the plant or their exudation by the roots is poorly understood. This knowledge is important for understanding the ecological adaptation of *Artemisia* and *Hypericum* species to different environments and for developing new cultivation technologies for these species that will combine high yield and high accumulation of biologically active and medicinally valuable compounds in the plant biomass.

One environmental stress that is common in various ecological niches is nutrient deficiency. Thus, all plant taxa, including those that synthesize specific classes of secondary metabolites, have to evolve mechanisms that will allow adaptations to fluctuating nutrition. This suggests that different classes of secondary compounds might play different roles in plant adaptations to nutrient deficiencies. Nitrogen (N), phosphorus (P), and iron (Fe) deficiency are the primary constraints on plant growth in nature and in the field (Elser et al., 2007; Pii et al., 2015). Our knowledge of the nutrient modulation of plant growth and secondary metabolism is based mostly on studies with *Arabidopsis* and a few crop plants, but most studies indicate an involvement of complex mechanisms of plant responses to nutrient availability, including sensing of the local levels of key nutrients by roots and sensing of the total nutrient requirements for plant growth (Jia et al., 2022). Further investigations of new crops, such as medicinal plants, are required in this field.

Several experiments have shown that nutrient deficiency can increase the accumulation of secondary compounds in both roots and aboveground parts of plants. For example, N deficiency increased the accumulation of flavonoids in the roots of tea and rapeseed plants (Liu et al., 2021; Shen et al., 2022), as well as in the aboveground parts of *Arabidopsis*, tomato, and carpetgrass (*Axonopus compressus*) (Stewart et al., 2001; Lea et al., 2007; He et al., 2021). Phosphate deficiency increased flavonoid accumulation in cluster roots in white lupin (Xiong et al., 2022) and in the leaves of tomatoes (Stewart et al., 2001). Iron deficiency increased the accumulation of secondary compounds in roots and leaves in dill (Wasli et al., 2018).

Nutrient deficiency can also enhance secretion of secondary compounds into the rhizosphere. For instance, N deficiency in legumes can induce root exudation of flavonoids, which initiate symbiosis with nitrogen-fixing rhizobia (Bais et al., 2006). Similarly, P deficiency can enhance the exudation of strigolactones by roots, which stimulate the branching and growth of mycorrhizal fungal hyphae, thereby increasing the probability of symbiosis between plants and mycorrhiza (Czarnecki et al., 2013) and aiding phosphate acquisition. In *Beta vulgaris*, Fe deficiency can increase flavin exudation, which can also improve Fe availability for plants (Sisó-Terraza et al., 2016). Despite numerous examples of the stimulatory effects of nutrient deficiency on the accumulation and the exudation of secondary compounds, the regulation of these processes in medicinal plants, such as *A. annua* and *H. perforatum*, is still practically unknown.

In nature, nutrients are often distributed heterogeneously in soil, leading to the evolution of complex mechanisms in plants that increase nutrient uptake efficiency. One such mechanism is the stimulation of lateral root proliferation into soil patches with high nutrient content, which is regulated by a combination of local and systemic signals (Jia et al., 2022). Split-root experiments have shown that the long-distance transport of systemic signals promote lateral root proliferation into the high-nutrient compartments (Drew, 1975; Bellegarde et al., 2017). In the field, local nutrient deficiency can induce the accumulation of secondary compounds without restricting plant growth, as overall nutrient demand is satisfied by the portion of roots in nutrient-sufficient patches. Therefore, we hypothesize that a local nutrient deficiency would induce the accumulation of secondary compounds without compromising plant growth.

In contrast to lateral root proliferation, which is regulated by both systemic and local signals in split-root experiments, the length of the root hairs is primarily regulated by a local signal (Bhosale et al., 2018; Giri et al., 2018), and root hairs elongate in the roots exposed to low levels of nutrient supply. Root hairs have been suggested to contribute to the accumulation of secondary compounds in the roots by serving as “factories” for their production. This is supported by studies on “hairy root” cultures, which have shown a significant accumulation of secondary compounds, including artemisinin (Wang et al., 2001; Liu et al., 2002). This high accumulation of artemisinin in hairy root cultures can be explained by considering that root hairs are analogs of leaf trichomes (Kellogg, 2001; Dayan and Duke, 2003), which are major sites of artemisinin accumulation (Ikram and Simonsen, 2017). However, further investigation is needed to determine whether root hairs can contribute to the accumulation of secondary compounds in intact plants and whether root hairs enhance the exudation of secondary compounds.

The aim of this study was to investigate how unequal distributions of N, P, and Fe affect plant growth, accumulation of artemisinin in plant biomass, and artemisinin exudation by roots in *A. annua*. We hypothesize that a heterogeneous nutrient supply would enhance the accumulation and exudation of valuable secondary compounds in important medicinal plants. We also evaluated the effect of heterogeneous nutrient distribution and nutrient deficiency on root hair formation, assuming that root hairs could modulate the accumulation of artemisinin in plant biomass and/or its exudation by roots. To account for potential species-specific responses to local nutrient deficiencies, we also tested the effect of a heterogeneous N supply on the accumulation of secondary compounds in biomass and their exudation by roots in *H. perforatum* plants.

## 2 Materials and methods

### 2.1 Experimental design overview for *Artemisia annua* and *Hypericum perforatum*

We used a split-root nutrition system for the two medicinal plant species to modulate the accumulation of secondary metabolites in biomass and their secretion as root exudates. For *A. annua*, half of the root system received a complete nutrient solution (NS) while the other half was supplied with a NS lacking either nitrogen (N), phosphorus (P), or iron (Fe). We assessed the impact of nutrient modulation on the accumulation of artemisinin in the leaves and roots and the exudation of artemisinin by the roots. For *H. perforatum*, we used the split-root nutrition system to investigate the effects of heterogeneous N supply on the accumulation of valuable secondary compounds in the shoot and roots and their exudation by the roots. To evaluate the impact of nutrient modulation on plant growth, we measured general plant growth traits for both medicinal species. Additionally, we investigated the effect of the different nutrient deficiencies on root hair development by measuring root hair length and root hair density. During the entire cultivation period, the conditions in the growth chamber were maintained at a 16 h/8 h day/night photoperiod (8:00–24:00 light), 22°C/18°C day/night temperature, 60/80% day/night air humidity, atmospheric CO_2_ concentrations, and a photosynthetically active radiation intensity of 190 µmol m^-2^ s^-1^.

### 2.2 Growth and experimental setup for *Artemisia annua*

Seeds of *A. annua* were obtained from Anamed International and were sterilized prior to sowing by incubating in 70% ethanol for 2 min, then in 2% sodium hypochlorite (2% commercial bleach) for 10 min, and then they were washed in deionized water. The seeds were sown between two irrigation mats with a complete modified NS based on Hoagland and Arnon’s recipe (Hoagland and Arnon, 1950). The full NS contained 5 mM Ca(NO_3_)_2_, 0.25 mM KH_2_PO_4_, 1.25 mM MgSO_4_, 1.75 mM K_2_SO_4_, and micronutrients 20 µM Fe, 1.25 µM Mn, 1.5 µM Zn, 25 µM B, 0.5 µM Cu, and 0.175 µM Mo. To avoid the negative effects of autotoxic and growth-inhibiting substances produced by *A. annua* itself, a continuous circular flux of NS was provided from the top by drippers fixed between the plates. Once the roots of the seedlings reached a length of 5 cm, they were transferred to 2.4 L hydroponic pots with 1.8 L NS (pH 5.5–6.0). Seven seedlings of the same height were transplanted into each pot. The NS was changed once per week. Thirty days after sowing (DAS), one group of plants was transferred into a self-built split-root nutrient hydroponics setup, while the remaining plants were kept in the control hydroponics system at one plant per pot as an additional control.

The self-built split-root hydroponic nutrient system used in this study consisted of two rectangular plastic containers (1.2 L; 16 cm × 12.5 cm × 9 cm) glued together, and the walls were hollowed out in the middle 2 cm deep and 2.5 cm wide (**Figure S1A**). Tight-fitting plastic lids were placed on the top of the containers, with a 2.5 cm diameter hole for the plant basket. At 30 DAS, the primary root of the *A. annua* plants was cut with a sterile scalpel, leaving 4 secondary root branches about 10 cm in length (**Figure S1B**). The *A. annua* plant was stabilized with a sponge in the pot, and two of the secondary root branches were directed to one of the compartments with the desired nutrient treatment.

To protect the NS and roots from light, the containers and lids were covered with light-impermeable white foil. The control plants were supplied with a full NS in both compartments, while the treatment plants received the full NS in one compartment and a deficient solution lacking nitrogen (N), phosphorus (P) or iron (Fe)) in the other compartment. The deficient solutions were prepared by omitting Ca(NO_3_)_2_ and replacing it with 0.8 mM CaSO_4_ for the minus-N treatment, omitting KH_2_PO_4_ for the minus-P treatment, and omitting Fe(III)-EDTA for the minus-Fe treatment. In the split-root hydroponic system, four replicates were used for each treatment. Additionally, an extra control with four treatment replicates of full nutrients and lack of N, P, or Fe was set up in a hydroponics system consisting of 2.4 L light-impermeable pots, with the whole root was preserved and placed in 1.8 L of NS. The pH of the NS was regularly monitored and controlled between pH 5.5 and 6.3. All treatments received constant aeration.

### 2.3 Growth and experimental setup for *Hypericum perforatum*

*Hypericum perforatum* L. (St. John’s wort) seeds were purchased from Rarexoticseeds (https://www.rarexoticseeds.com/) and were sterilized with 2.5% sodium hypochlorite for 10 min before being washed thoroughly with deionized water five times. One sterilized *H. perforatum* seed was germinated in rockwool cubes (36 × 36 × 36 mm), which were soaked in a 10% full NS containing 500 µM KNO_3_. The full NS contained 1 mM CaSO_4_, 1 mM K_2_HPO_4_, 1 mM KH_2_PO_4_, 2 mM MgSO_4_ (Paponov et al., 2021a) and micronutrients including 15 µM Fe, 10 µM Mn, 5 µM Zn, 30 µM B, 0.75 µM Cu, and 0.5 µM Mo.

At 35 DAS, the lower section of the primary root was removed by making two inclined cuts to form a cleft in the rockwool cube (**Figure S1C**), while maintaining the upper (lateral) roots growing toward both sides. At 50 DAS, when the plants had reached a size of 15 cm, the plant with the cube was fixed onto the lid of a self-built, high-pressure aeroponics system (**Figure S1D**) consisting of 2 transparent plastic containers (16 × 12.5 × 9 cm) glued together by the sides.

The plant was positioned in the middle of the lid, exactly over the walls of the two connected containers, so that half the roots were growing in each container (**Figure S1E**). The containers had holes in the bottom for drainage and in the side wall for a mist nozzle (0.5 cm), which was directed toward the root of the plant. The mist nozzle produced mist using high-pressure aeroponics for 4 s with an interval of 3 or 4 min, controlled by a DCB01 asymmetrical recycler timer (Carlo Gavazzi, Schneider Electric, Denmark). Six control plants received the full NS containing 5 mM KNO_3_, while six treatment plants received full NS in one container and NS lacking KNO_3_ in the other container for 26 days until final harvest. The pH of the NS was monitored regularly and controlled between pH 6.3–6.9.

### 2.4 Root exudate extraction for *Artemisia annua* and *Hypericum perforatum*

One day prior to the final plant sampling, the root exudates were collected by incubation of the root parts in the two-compartment setup described above (section 2.2), covered by light-impermeable foil, and containing 950 mL continuously aerated deionized water with a pH of 6.0. The roots were incubated for 24 h (*A. annua*) or 16 h (*H. perforatum*).

The deionized water containing root exudates was prefiltered using a Sigma-Aldrich® vacuum filtration assembly (Z290432-1EA, Merck, Darmstadt, Germany) and Nalgene bottle-top sterile filters (45 mm diameter and 0.45 μm pore size) (Z370533, Merck, Darmstadt, Germany). Approximately 800–1700 mL (*A. annua*) and 850 mL (*H. perforatum*) deionized water-exudate solution were loaded onto Bond Elut™ C18 (Agilent Technologies, Santa Clara, CA, USA) solid-phase extraction cartridges with a 1 g bed mass and 40 µm particle size to trap the non-polar and semi-polar secondary compounds. Columns were activated with 1 mL 100% MeOH (10516279, Fisher Scientific, Waltham, MA, USA) followed by 1 mL 1% aqueous formic acid (33015, Fluka, Honeywell, Morris Plains, NJ, USA). The columns were washed with 2 mL deionized water, and the hydrophobic compounds were eluted with 1 mL of 2% formic acid in MeOH. The eluent was stored at −20°C. The secondary compounds in the root exudate extracts from *A. annua* and *H. perforatum* were measured by ultra-high-performance liquid chromatography (UHPLC).

### 2.5 Final sampling of *Artemisia annua*

Roots of *A. annua* plants were transferred into deionized water for 24 h for the collection of root exudates. Depending on the treatment, each plant was transported to one pot or two-compartment setups covered with light-impermeable foil and containing 1900 or 950 mL, and continuously aerated in deionized water with a pH of 6.0. At the final harvest at 51 DAS, the *A. annua* leaf, shoot, root, and corresponding root halves were weighed and sampled. The total dry biomass, leaf dry matter percentage (LDM(%)), stem dry matter (SDM(%)), root dry matter percentage (RDM(%)), and ratio of root weight to total plant weight (RWR) were determined. About 1 g root and 1 g leaf material were collected for each treatment, immediately frozen in liquid nitrogen, and stored at -80°C. A further 1 g of root material was collected, weighed, and preserved in 50% ethanol for analysis of root hairs.

### 2.6 Final sampling of *Hypericum perforatum*

At 76 DAS, the total fresh weight of the leaf, stem, and root of *H. perforatum* was recorded, and an aliquot of about 1 g fresh leaf and root material was weighed, immediately frozen in liquid nitrogen in a pre-weighed 5 ml Eppendorf tube, and vacuum lyophilized for 36 (leaves) and 24 (roots) h in a BK-FD10S freeze-dryer (BIOBASE, Jinan, China). After determining the dry matter % of the samples, the leaf and root material were powdered (Star-Beater VWR with 5 mm metal balls, 29 Hz for 3 min) to fine dust and stored at −80°C until further processing UHPLC analysis.

The total dry biomass, LDM(%), SDM(%), RDM(%), and RWR were determined. A further 0.3–0.5 g of root material was collected, weighed, and preserved in 50% ethanol for analysis of root hairs.

### 2.7 Analysis of roots and root hairs for *Artemisia annua* and *Hypericum perforatum*

At the final harvest, a representative aliquot of 1–2 g root per treatment was conserved in 50% ethanol for root hair length and root hair density measurements at a microscope. The root samples were then mounted in water and visualized with an Olympus CX-41 microscope (Olympus Corporation, Tokyo, Japan) and dark-field illumination. Images were captured with an ocular-mounted Toupcam U3CMOS 5.1 MP camera (ToupTek Europe, Stansfield, United Kingdom) using ToupView 3.7 software. The average density (hairs/mm) and length (mm) of the root hairs were determined using Fiji software. The dataset is based on 10 measurements of representative root segments of 3 plant replications per treatment.

For *H. perforatum* 10 randomly chosen recordings of root segments per plant were investigated for root hair density and root hair length measurement, resulting in investigations of 59–60 segments per treatment and until 12 root hair length measurements per segment.

### 2.8 Sample preparation for UHPLC analysis of *Artemisia annua* and *Hypericum perforatum* in leaves and roots

Lyophilized and powdered leaf and root samples of *A. annua* (50 mg) and *H. perforatum* (100 mg) prepared by treatment at 29 Hz for 3 min (Starbeater, VWR, Radnor, PA, USA) were vortexed with a 5 mm in diameter steel bead and 1.5 mL 80% methanol for 20 min at maximal speed in 2 mL Eppendorf tubes. The extract was then centrifuged for 7 min at 17,000 × g (MicroStar 17R, VWR, USA), and the supernatant was collected. The supernatant was centrifuged again to prevent later sedimentation. The clean supernatant was stored at −20°C until analysis by UHPLC.

### 2.9 Quantitative determination of artemisinin

Artemisinin was quantified using UHPLC-MS/MS analysis performed on an Agilent 1290 LC system (Agilent, Waldbronn, Germany) connected to an API 3200 triple quadrupole mass spectrometer by a TurboIon source (AB Sciex, Darmstadt, Germany). Artemisinin was separated on a Nucleoshell C18 column (2.6 µm, 50 x 3 mm; Macherey-Nagel, Düren, Germany) at a flow rate of 500 µl min^-1^ using 0.02% (v/v) acetic acid in water or in acetonitrile as eluents A and B, respectively. The ion source was operated in the positive mode, and data were acquired in the multiple reaction monitoring mode with target scan time of 50 ms. The IntelliQuant algorithm of the Analyst 1.6.2 software (AB Sciex, Darmstadt, Germany) was used to integrate the peaks for artemisinin. Concentrations were calculated using an artemisinin standard curve in the range of 0 to 5.6 µg ml^-1^ and divided by the dry weights. Our full protocol for determining artemisinin level in *A. annua* was recently published (Paponov et al., 2023).

### 2.10 Quantitative determination of secondary compounds of *Hypericum perforatum* by UHPLC

The secondary compounds were analyzed by UHPLC (1290 Infinity II, Agilent Tech-nologies, Santa Clara, CA, USA) with a diode array detector and an electrospray ionization single-quadrupole detector (6120 SQ, Agilent Technologies, Santa Clara, CA, USA). Separation was achieved on an Ascentis Express C18-column (100 × 2.1 mm, 2 µm, Supelco, Merck, Darmstadt, Germany). Quantifications were made based on the UV-Vis absorbance at the detection windows of 280, 320, 360, and 590 nm for catechins, hydroxycinnamic acids/APG, flavonols, and NDAs, respectively. Additional details of the protocol can be found in our recent paper (Paponov et al., 2023).

### 2.11 Statistic

Data were statistically analyzed by analysis of variance (one-way ANOVA). When significant treatment effects were indicated by ANOVA, Fisher’s protected LSD test was used to compare the individual means (Statistica 13 software package, Palo Alto, CA, USA).

## 3 Results

### 3.1 Plant growth and dry matter allocation in *Artemisia annua*

**Figure 1:**
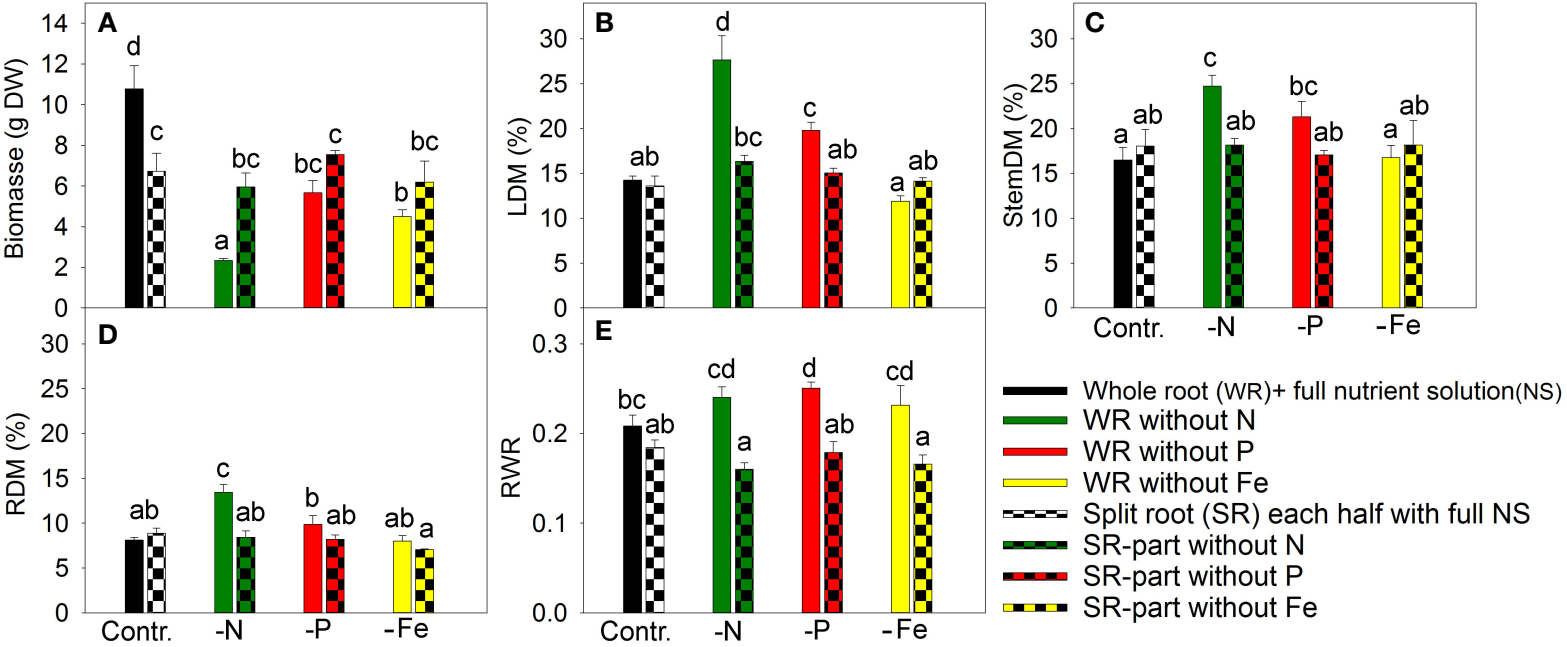
The effect of nitrogen (N), phosphorus (P), and iron (Fe) deficiency and heterogeneous N, P, and Fe supply using a split-root system on plant biomass **(A)**, leaf dry matter percentage (LDM (%)) **(B)**, stem dry matter percentage **(C)**, root dry matter percentage (RDM (%)) **(D)**, and ratio of root weight to total plant weight (RWR) in **(E)** *Artemisia annua* (*n*=4). Differences between means with different letters are statistically significant at p<0.05.

In a nutrient exclusion experiment with *A. annua*, N deficiency caused the most significant reduction in plant growth, resulting in a 4.6-fold reduction in plant biomass (**Figure 1A**). This is attributed to the high N demand in plant tissues for unlimited growth and the limited pool of available “storage” N for re-utilization.

Exclusion of P from the NS for 20 days led to a 1.9-fold reduction in plant biomass, while Fe exclusion caused similar plant growth inhibition (**Figure 1A**) despite lower Fe demand. Fe has low mobility in the phloem (Marschner, 1995); therefore, plants show chlorosis and reduced photosynthesis in the upper leaves following disruption of the direct supply of Fe from the roots.

In split-root systems, half of the roots were grown in nutrient-deficient solution and the other half in full NS. The split-root system was established by cutting the primary root to induce a strong outgrowth of lateral roots, which were then distributed between two merged pots. The root cutting and cultivation in this system decreased the plant growth under non-limiting nutrient supply compared with intact plants (**Figure 1A**). The exclusion of a nutrient from one part of the roots did not have any inhibitory effect on plant growth compared to the split-root control (**Figure 1A**), suggesting that supplying nutrients to only half of the root system was sufficient to meet the plant’s nutrient demand.

Leaf dry matter increased under total N and P deficiency (**Figure 1B**), which might reflect the accumulation of starch due to a stronger inhibition of plant growth (e.g., cell elongation and cell division) than photosynthesis (Qiu and Israel, 1992; Paponov and Engels, 2003; Paponov et al., 2021b). However, Fe deficiency did not enhance leaf dry matter content despite its inhibitory effect on plant growth. This indicated that the primary target of Fe deficiency is photosynthesis, which is related to the loss of chlorophyll, impairment of photosynthetic electron transport, and lower content of starch and sugars, as previously shown (Arulanantham et al., 1990; Abadia, 1992). The plants grown under heterogeneous nutrient supply had similar dry matter percentages in leaves as plants grown with a whole root system under full nutrient supply (**Figure 1B**), indicating that growth and photosynthesis were balanced in these plants. The effects of nutrient deficiency and split-root treatments on dry matter content in the stem and roots were similar to their effects on leaf dry matter content (**Figures 1C, D**).

Dry matter allocation to roots was sensitive to nutrient deficiency, as complete exclusion of any studied element increased or tended to increase root dry matter allocation (**Figure 1E**). In contrast, supplying a nutrient-deficient solution to half of the root system did not affect dry matter allocation between the roots and shoots (**Figure 1E**). Heterogeneous nutrient supply did not result in nutrient limitation at the whole-plant level.

### 3.2 Artemisinin accumulation in leaves and roots

**Figure 2:**
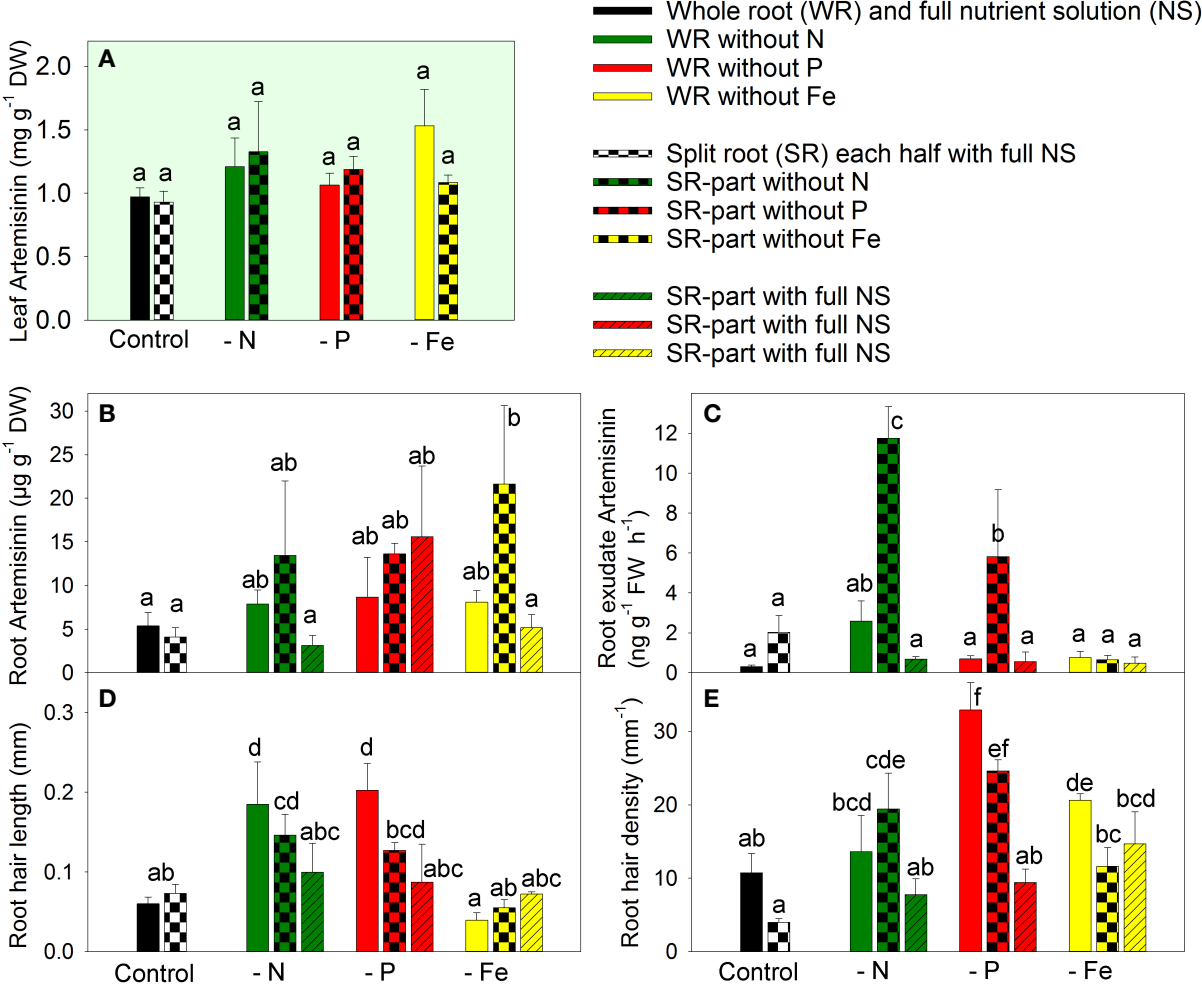
The effect of nitrogen (N), phosphorus (P), and iron (Fe) deficiency and heterogeneous N, P, and Fe supply using a split-root system on artemisinin (AN) concentration in leaves **(A)**, AN concentration in roots **(B)**, the rate of AN exudation from roots **(C)**, the length of root hairs **(D)**, and root hair density **(E)** in *Artemisia annua* (*n*=4). Differences between means with different letters are statistically significant at p<0.05.

The concentration of artemisinin in the leaves remained unchanged regardless of the modulation of nutrient supply through nutrient deficiency or split-root supply (**Figure 2A**). Despite the significant biomass reduction observed in response to nutrient deficiency, there was no significant increase in artemisinin concentration in the leaves, indicating a parallel inhibition of both biomass accumulation and artemisinin biosynthesis.

Artemisinin concentration in the roots was much lower compared to the leaves (**Figures 2B** vs. **2A**). Complete exclusion of nutrients or local exclusion of N or P in the split-root system did not affect artemisinin accumulation in the roots. However, local Fe exclusion in the split-root system led to increased artemisinin accumulation in the Fe-deficient root part. This suggests the involvement of both local and systemic signals in the regulation of artemisinin accumulation in *A. annua* roots under heterogeneous Fe supply. The fact that Fe deficiency enhanced artemisinin accumulation only when Fe was sufficiently available through another part of the root system suggests that the regulation of artemisinin accumulation is influenced by systemic signals.

### 3.3 Artemisinin exudation by the roots

The pattern of artemisinin exudation by the roots differed from that of artemisinin accumulation in the roots, indicating that exudation is regulated by a specific mechanism rather than the simple diffusion of artemisinin from the root tissue. The complete exclusion of any essential element did not significantly modulate artemisinin exudation (**Figure 2C**). However, the split-root system, which resulted in local exclusion of N or P from one part of the roots, strongly increased artemisinin exudation by 5.8-fold and 2.9-fold, respectively, from the nutrient-deficient root parts. The upregulation of artemisinin exudation occurred only when the local deficiency was combined with a sufficient nutrient supply in the whole plants, indicating that both local and systemic signals are involved in the regulation of artemisinin exudation under heterogeneous N and P supply.

The supply of N, P, or Fe had varying effects on root hair formation. The complete exclusion of N and P increased root hair length by 3.1-fold and 3.4-fold, respectively, whereas Fe deficiency did not affect root hair length (**Figure 2D**). The local exclusion of N and P also increased or tended to increase root hair length, but to a lesser extent than complete nutrient deficiency. The root hair length was the similar in the local rich nutrition zone compared to the plants grown in the full NS. These observations suggest that local signals predominantly regulate root hair length under heterogeneous nutrient supply.

### 3.4 Root hair formation

The greatest root hair density was found following the complete exclusion of phosphate from the NS. Similarly, local phosphate deficiency also increased root hair density (**Figure 2E**). The other nutrient deficiencies also increased (Fe) or tended to increase (N) the root hair density. No significant differences were detected in root hair densities between roots cultivated with local or whole-plant N or P deficiencies, and no significant differences were noted in root hair densities between roots cultivated at locally sufficient and equally sufficient nutrient supply in split-root plants. These findings indicate that root hair density was regulated predominantly by local signals. The different responses of biomass artemisinin accumulation, root hair formation, and artemisinin exudation by roots indicated that artemisinin accumulation/exudation and root hair formation were regulated by different mechanisms.

### 3.5 Hypericum perforatum

In the experiment with *H. perforatum*, we investigated only the effect of a split-root supply of nitrate on the growth and accumulation of biologically active compounds. The split-root system had no effect on the growth and growth-related traits of *H. perforatum* plants (**Table 1**), consistent with the results obtained for *A. annua* plants. However, the heterogeneous N supply enhanced or tended to enhance the concentration of hypericin, catechin, and rutin isomer by 59%, 10%, and 10%, respectively, in *H. perforatum* leaves, indicating a potential positive effect of the split-root system on the accumulation of valuable compounds (**Table 2**). In the roots, the heterogeneous N supply decreased the accumulation of catechin by 10% and increased the accumulation of other naphthodianthrones (NDAs) by 41%. No difference was found between the two halves of the split-root systems supplied with full or N-deficient NS (**Table 2**).

**Table 1:** The effect of heterogeneous nitrogen (N) supply using the split-root system on plant biomass; dry matter content in leaves, stem, and roots; and root weight ratio (RWR) in *Hyperi-cum perforatum* (*n*=6).

Root system	Whole root	Split root		1-way ANOVA
Nitrogen levels	High	High	Low	
Plant parameters				
Biomasse (g DW)	3.5 ± 0.4	3.2 ± 0.3		NS
Leaf DM (%)	18.6 ± 0.3	18.9 ± 0.3		NS
Stem DM (%)	16.8± 0.7	15.8 ± 0.7		NS
Root DM (%)	5.3 ± 0.4	5.5 ± 0.4	5.9 ± 0.3	NS
RWR	0.18 ± 0.008	0.22 ± 0.002		NS

**Table 2:** The effect of heterogeneous nitrogen (nitrate) supply using a split-root system on leaf and root contents and rates of root exudation of biologically active compounds in *Hypericum perforatum* cultivated in an aeroponics system (*n*=6).

Biologically active compounds	Control	Split-root (N rich)	Split-root (N poor)	*p*-value ANOVA of individual compounds
LEAF (mg g^-1^ DW)				
Naphthodianthrones				
Hypericin	0.17 ± 0.03	0.27 ± 0.05		NS, p=0.08
Pseudohypericin	0.12 ± 0.02	0.13 ± 0.01		NS
Flavan-3-ols				
Epicatechin	4.9 ± 0.1	5.2 ± 0.1		NS, p=0.069
Catechin	26.5 ± 0.7 (a)	29.0 ± 0.4 (b)		** p=0.009
Procyanidin dimer	6.7 ± 0.2	6.7 ± 0.2		NS
Phenolic acids				
Chlorogenic	23.5 ± 0.8	23.4 ± 0.3		NS
Coumaroylquinic	18.0 ± 1.7	15.5 ± 1.0		NS
Flavonols				
Kaempferol-glycoside	12.7 ± 1.0	14.6 ± 0.4		NS
Rutin isomer	8.7 ± 0.4 (a)	9.6 ± 0.2 (b)		* p=0.04
Rutin	11.6 ± 0.6	11.7 ± 0.7		NS
Que-isomer	9.2 ± 0.4	9.6 ± 0.4		NS
Que3glc	6.2 ± 0.8	7.3 ± 0.3		NS
Quercetin	5.2 ± 0.3	5.5 ± 0.2		NS
ROOT (µg g^-1^ DW)				
(Epi)Catechin	10.2 ± 0.3 (b)	9.1 ± 0.3 (a)	9.3 ± 0.4 (ab)	NS, p= 0.07
Other NDAs	0.54 ± 0.0 (a)	0.73 ± 0.1 (ab)	0.79 ± 0.1 (b)	NS, p= 0.09
ROOTEXUDATE (nmol g^-1^ FW h^-1^)				
Hypericin	4.2 ± 0.8 (a)	10.8 ± 2.6 (b)	8.8 ± 1.7 (ab)	NS, p= 0.06
Other NDAs	24.9 ± 9.2	25.9 ± 8.6	11.6 ± 2.2	NS
SUM Hypericin + NDAs	29.1 ± 9.9	36.6 ± 10.5	20.4 ± 3.5	NS

Values are means ± SE. The data were analyzed using LSD test. Significant effects are labeled with letters at the 0.05 probability level. naphtodianthrone (NDA). One-way ANOVA is presented on the right.

Under heterogeneous N supply, the rate of hypericin exudation in both N-rich and N-deficient parts of roots increased by 2.6-fold and 2.1-fold, respectively. However, the increased hypericin exudation in the N-deficient part was accompanied by a reduced exudation of other NDAs, compared to the plants supplied with sufficient N (**Table 2**). Thus, local N deficiency did not appear to stimulate the exudation of a valuable compound from *H. perforatum* plants.

## 4 Discussion

Plants have evolved the ability to optimize their resource allocation by adjusting their lateral root formation in response to heterogeneous nutrient distribution in the soil, thereby promoting root growth into nutrient-rich soil patches (Oldroyd and Leyser, 2020). Our study shows that plants also use local and systemic signals to differentially regulate the root exudation of secondary compounds in response to heterogeneous nutrient availability. Specifically, we found that *A. annua* plants exhibited differential adjustment of their root exudation of artemisinin when exposed to heterogeneous N and P distribution.

The evidence that exudation of biologically active compounds is regulated differentially in nutrient-rich and nutrient-poor locations is based on split-root experiments showing that exudation of artemisinin increased 5.8-fold and 2.9-fold, respectively, in the parts of *A. annua* roots exposed to local N or P deficiencies. The involvement of both local and systemic signals in the regulation of artemisinin exudation was supported by the greater exudation from plants growing under local N or P deficiency than under complete N deficiency or under full nutrient supply. This means that a combination of nutrient-low local signals and nutrient-rich systemic signals is required for strong enhancement of the exudation of artemisinin from roots under a heterogeneous nutrient supply. This enhancement of root exudation under local nutrient deficiency is opposite to the response of lateral roots, which proliferate in nutrient-rich patches (Oldroyd and Leyser, 2020), indicating that different mechanisms may regulate these responses. However, we cannot exclude the possibility that some molecular players involved in the regulation of root proliferation under a heterogeneous nutrient supply might also be involved in the differential regulation of secondary compound exudation by roots.

The root exudation of biologically active compounds in response to heterogeneous N supply differed between *H. perforatum* and *A. annua*, as indicated by a tendency for increased exudation of secondary compounds in the N-rich part of roots in *H. perforatum*. Heterogeneous N supply also had a different effect on the accumulation of medicinally valuable compounds in the aboveground parts, as indicated by increases in the accumulation of hypericin, catechin, and rutin isomers in *H. perforatum* leaves. Thus, a heterogeneous N supply can be a promising tool to enhance the phytochemical quality of H. perforatum plants. The different patterns of responses of accumulation and exudation of biologically active compounds between *A. annua* and *H. perforatum* indicate that the evolved mechanisms regulating secondary compound homeostasis might be species- and compound-specific.

### 4.1 Modulation of artemisinin exudation is unrelated to the formation of root hairs

One factor that can affect the amount of root exudation is root hair formation, as demonstrated in a previous investigation showing that the absence of root hairs in a barley mutant strongly reduced total root exudation (Holz et al., 2018). However, the specific effects of root hairs on the secretion of secondary compounds are unknown. In our study, the pattern of the response of root hair formation (characterized by root hair length and root hair density) to a modulated nutrient supply differed from the pattern of artemisinin exudation, indicating that different mechanisms are involved in the regulation of these two processes. In contrast to the regulation of artemisinin exudation, where both systemic and local signals are involved, the root hair length and density appeared to be predominantly regulated by a local signal. The evidence for a predominant role of a local signal is based on the observation that root hair length (RHL) under low local deficiency was similar to the RHL under low homogenous deficiency and that RHL under local high nutrient supply was similar to RHL of roots grown at high homogenous nutrient supply (**Figure 2D**). This conclusion for a dominant regulation of RHL by a local signal under heterogeneous nutrient supply is in agreement with the conclusion about regulation of RHL under local P availability based on the responses of *Arabidopsis* and rice to low external phosphate (Bhosale et al., 2018; Giri et al., 2018).

### 4.2 Ecological benefits of increased exudation of secondary compounds in local nutrient-deficient patches

Our observation that the exudation of secondary compounds by roots is regulated differently in different species might be related to the specific effects of these compounds on major root-to-soil processes, particularly nutrient acquisition. In *A. annua*, the pattern of artemisinin exudation was opposite to the pattern of lateral root proliferation (Oldroyd and Leyser, 2020), suggesting a different impact of these root traits on the major processes that define nutrient acquisition. Lateral root proliferation provides maximal benefits if lateral roots proliferate in nutrient-rich patches because lateral root exploration of the soil patches will enhance nutrient movement to the root surface both by transpiration-driven mass flow and diffusion (Nye, 1977). The different responses of artemisinin exudation might be related to its different functions, as artemisinin exudation does not affect nutrient acquisition directly but instead modulates the availability of nutrients through interactions with other organisms in the rhizosphere. These interactions can include interactions with neighboring plants, bacteria, and mycorrhizae.

Several investigations have shown that artemisinin has herbicidal activity on plants and can inhibit the growth of different plant species, including lettuce, barley, ray, radish, and others [reviewed in (Jessing et al., 2014)]. Therefore, we suggest that an enhancement of artemisinin exudation in locally nutrient-deficient patches could inhibit the root growth of neighboring plants. The role of secondary compounds with allelopathic activity in root exudates has also been well characterized in other plants. For example, wheat (*Triticum aestivum* L.) and rice (*Oryza sativa* L.) were able to release chemicals to inhibit the growth of weeds (Yang and Kong, 2017; Kong et al., 2018).

The enhanced exudation of artemisinin in nutrient-poor patches of soil will reduce the competition for nutrient acquisition with neighboring plants; however, whether this function brings significant benefits to *A. annua* plants is not clear because of the low level of available nutrients in these patches. Therefore, we assume that differential exudation would also modulate the activity of other organisms that can enhance the available levels of essential nutrients, such as N and P. For example, root exudates could stimulate positive interactions with soil microbes that contribute to plant nutrient absorption, stress tolerance, and pathogen defense (Sasse et al., 2018; Ma et al., 2022; Pantigoso et al., 2023).

Under N deficiency, increased exudation of flavonoids is generally observed in both legumes (Liu and Murray, 2016) and non-legumes (Gough et al., 1997; Webster et al., 1998) that facilitate root interactions with N-fixing bacteria. In legumes, flavonoids facilitate rhizobial symbiosis by activating the rhizobial nodulation genes (nod), thereby modulating the bacterial release of nod factors that lead to plant nodule development essential for endosymbiotic bacteria and N fixation (Liu and Murray, 2016). In addition to interacting with diazotrophs, certain plant species can also modulate the N cycle in soils by either suppressing biological nitrification or denitrification via root exudation, thereby promoting the retention of more N in the soil for plant uptake (Coskun et al., 2017).

Under P deficiency, plants use different mechanisms to increase P uptake, including symbiosis with mycorrhizal fungi (Ryan et al., 2012; Nazeri et al., 2014; Wen et al., 2019). Mycorrhization is regulated by root exudates, with the signaling compound strigolactone playing a key role. P deficiency enhances the exudation of strigolactone due to the upregulation of the G-type ABC transporter PDR1 (Kretzschmar et al., 2012). The exudation of other secondary compounds, such as phenolics, can also suppress microbial communities that compete with plant roots for limited available P in the rhizosphere (Weisskopf et al., 2006). How the exudation of artemisinin and other secondary compounds from *A. annua* roots affects mycorrhizal inoculation is unknown and requires further investigation.

## 5 Outlook

Our study provides new insights into the regulation of root exudation and secondary metabolite accumulation in plants. *A. annua* plants are able to sense both whole plant and local nutrient levels, indicating the involvement of local and systemic signals in regulating root exudation of artemisinin. The finding that split-root nutrition can enhance the accumulation of secondary compounds in *H. perforatum* suggests that this technique holds promise for the cultivation of crops with high-value metabolites. Understanding the complex network of interactions between secondary compounds and primary metabolism, ROS, plant hormones, and other players is crucial for understanding plant ecology and developing technologies for efficient production of medicinal compounds. Further research should investigate these mechanisms in other plant species and explore the ecological benefits of this root exudation response and accumulation secondary compounds in plant biomass.

## Data availability statement

The raw data supporting the conclusions of this article will be made available by the authors, without undue reservation.

## Author contributions

Conceptualization, IP; methodology, IP, validation, MP and IP; investigation, JF, MP, IP, and JZ; resources, IP and CL; data curation, JF and MP; writing—original draft preparation, MP and IP; writing–review and editing, IP, MP, CL, and JZ; supervision, IP and CL; project administration, IP, funding acquisition, IP. All authors contributed to the article and approved the submitted version.
