# Supplementary material for: Heterogeneous nutrient supply modulates root exudation and accumulation of medicinally valuable compounds in Artemisia annua and Hypericum perforatum
Source: Front Plant Sci. 2023 Jun 2;14:1174151. doi: 10.3389/fpls.2023.1174151 (PMC10272524; doi:10.3389/fpls.2023.1174151)
Supplement: Supplementary file 1 [file DataSheet_1.pdf]

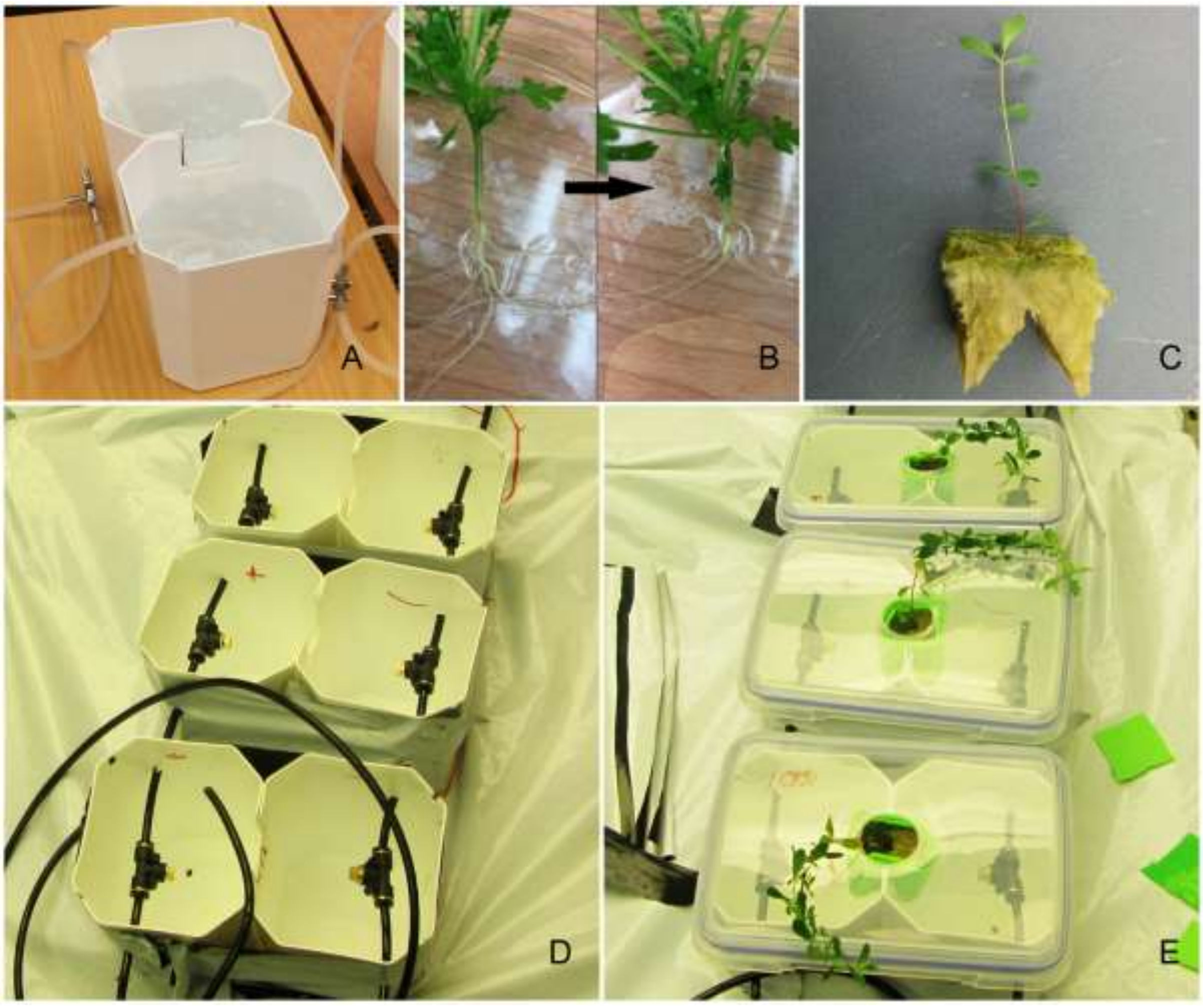

Figure S1. Experimental setup for cultivation of *Artemisia annua* and *Hypericum perforatum*. A) The split-root hydroponic system used for cultivation of *A. annua* consisted of two rectangular plastic containers that were glued together. B) The primary root of the *A. annua* was cut with a sterilized scalpel, leaving four secondary root branches. C) At 35 DAS, the lower section of the primary root of *H. perforatum* was removed by making two inclined cuts to create a cleft in the rockwool cube. D) High-pressure aeroponics system for cultivation of *H. perforatum*. E) The *H. perforatum* plants were positioned in the middle of the aeroponics lid, directly over the walls of the two connected containers, with half of the roots growing in each container.
